# Amniotic Band Syndrome: A 14‐Year‐Old Follow‐Up Case Report

**DOI:** 10.1155/cro/4411786

**Published:** 2026-09-07

**Authors:** Mohammed Alsahil, Iftikhar Ahmed Mukhtar, Dana Al Omran, Ahmed Alkooheji, Adel Alshihri

**Affiliations:** ^1^ Orthopedic Department, Eastern Health Cluster, Dammam, Saudi Arabia; ^2^ Orthopedic Department, Royal Medical Services, Al Riffa, Bahrain, jrms.mil.jo; ^3^ Orthopedic Department, Riyadh First Health Cluster, Riyadh, Saudi Arabia

## Abstract

Streeter′s dysplasia, also known as amniotic band syndrome (ABS), is a rare congenital condition characterized by a wide spectrum of malformations, deformities, and, in severe cases, limb amputations. Other manifestations associated with ABS include clubfoot, distal finger or toe hypoplasia, and syndactyly. Although several theories have been proposed, the exact etiology of this syndrome remains unclear. In this case report, we present the 14‐year follow‐up of a patient diagnosed with ABS at birth who has been managed through a multidisciplinary approach since infancy. This report highlights the patient′s clinical progression over the years and emphasizes the importance of multidisciplinary collaboration in achieving favorable clinical and functional outcomes.

## 1. Introduction

Amniotic band syndrome (ABS), also known as Streeter′s dysplasia, is a rare congenital condition associated with a wide spectrum of clinical manifestations. Orthopedic abnormalities include upper and lower limb deformities, constriction rings, distal limb hypoplasia, syndactyly, clubfoot, and congenital amputations. Nonorthopedic abnormalities may also occur and involve the craniofacial region, trunk, and, less commonly, internal organs, resulting in considerable variability in clinical presentation [1–9].

Prenatal ultrasonography (US) may facilitate early diagnosis by demonstrating constriction bands or associated fetal abnormalities. However, diagnosis may be challenging, and some cases remain undetected until birth, particularly when the deformities are subtle or the amniotic bands are not clearly visualized [1, 4].

The reported incidence of ABS ranges from 1 in 1000 to 1 in 15,000 live births and approximately 178 per 10,000 spontaneous abortions, with no clear sex predominance reported in the literature [3, 5]. The exact etiology remains uncertain, and several theories have been proposed to explain the pathogenesis of ABS. Streeter′s intrinsic theory suggests that an underlying defect in embryonic development results in tissue disruption and the subsequent formation of constriction bands. In contrast, Torpin′s extrinsic theory proposes that early rupture of the amniotic sac leads to the formation of fibrous bands that entrap and compress developing fetal structures. Although neither theory fully explains all clinical presentations, the extrinsic theory remains the most widely accepted explanation in the current literature [2, 6, 8].

Given the broad clinical spectrum of ABS, careful evaluation for associated anomalies and individualized multidisciplinary management are essential. Management often requires collaboration among orthopedic surgeons, plastic surgeons, rehabilitation specialists, prosthetists, physiotherapists, and, when appropriate, psychological support to optimize functional outcomes and quality of life [8–10]. In this report, we present the 14‐year follow‐up of a patient with ABS, highlighting the long‐term clinical course and the importance of multidisciplinary care in achieving favorable clinical and functional outcomes.

## 2. Case Presentation

A 14‐year‐old girl was born prematurely at 30 weeks′ gestation to healthy parents who were second cousins. She was delivered via spontaneous vaginal delivery and was admitted to the Neonatal Intensive Care Unit (NICU) for 7 days before being transferred to the regular nursery and subsequently discharged home.

During early pregnancy, the mother was diagnosed with premature rupture of membranes and experienced intermittent vaginal bleeding throughout the pregnancy. She was followed up at the Obstetrics Clinic, where US revealed a constriction around the left leg. However, she could not recall the exact timing of the examination, and the report could not be retrieved from the medical records. The pregnancy was closely monitored, and no prenatal intervention was performed.

The family history was unremarkable. Both parents were nonsmokers, with no family history of similar conditions, congenital anomalies, or genetic disorders, and there was no known exposure to teratogenic substances during pregnancy.

At birth, physical examination revealed a left below‐knee amputation with a mild constriction ring around the left thigh. Additional findings included hypoplasia of the right foot toes, the first to third fingers of the right hand, and the third and fourth fingers of the left hand. The right foot demonstrated congenital talipes equinovarus (CTEV). These findings were confirmed by plain radiographs.

Genetic testing was unremarkable. The patient was subsequently followed by the general pediatrics and pediatric orthopedic teams for further evaluation of the congenital anomalies and their underlying etiology. A diagnosis of ABS was established. Management extended throughout childhood and adolescence and involved coordinated multidisciplinary care provided by pediatric orthopedic surgeons, rehabilitation specialists, therapists, orthotists, and social workers. The primary goals were to maximize functional independence through surgical stump revisions, prosthetic fitting, occupational and physical therapy, and psychological support while minimizing complications during growth.

### 2.1. Neonatal and Infant Management

During the neonatal period, management focused on the right lower limb clubfoot, monitoring the constriction rings on the left thigh and the proximal interphalangeal (PIP) joint of the left little finger, and excluding other associated congenital malformations. No immediate postnatal surgical intervention was required, as the left below‐knee stump had healed and the constriction rings were mild.

The right CTEV was managed using the Ponseti method with serial casting over 6–8 weeks to gradually correct the deformity. Once near‐neutral alignment was achieved, a nighttime foot abduction brace was prescribed to maintain the correction. Early management of the foot deformity facilitated subsequent standing and independent walking.

In parallel, the rehabilitation team and orthotists planned for the patient′s mobility needs related to the left below‐knee limb deficiency. At approximately 10 months of age, when she began attempting to pull to stand, she was fitted with her first left below‐knee prosthesis. She achieved supported standing at approximately 11 months of age and subsequently underwent physical therapy to improve balance and gait. The family was educated on proper prosthesis application and encouraged to promote its regular use during daily activities to facilitate functional adaptation.

### 2.2. Childhood and Adolescent Surgical Interventions

As the patient grew, she underwent three stump revision procedures at the ages of 3, 8, and 10 years to address osseous overgrowth of the left residual limb and relieve skin pressure caused by the prominent bone ends. Each procedure involved trimming and smoothing the overgrown tibia and fibula, debulking scar tissue when necessary, and ensuring adequate soft tissue and muscle coverage over the bone ends. All procedures healed uneventfully and improved the comfort of prosthetic use.

The patient also required multiple prosthetic replacements throughout childhood to accommodate growth. Ongoing rehabilitation and patient education were provided to maximize functional independence and optimize long‐term outcomes.

### 2.3. Current Status

At the age of 14 years, our patient is thriving and able to perform her daily activities independently. For mobilization, her left lower limb is fitted with a prosthesis, and she is able to walk without any difficulties or restrictions. She is also able to bear full weight on her right lower limb without difficulty.

Regarding upper limb function, the patient uses her left hand for most daily activities, including writing and handling objects. She is also able to untie knots and change her clothes independently.

**Figure 1:**
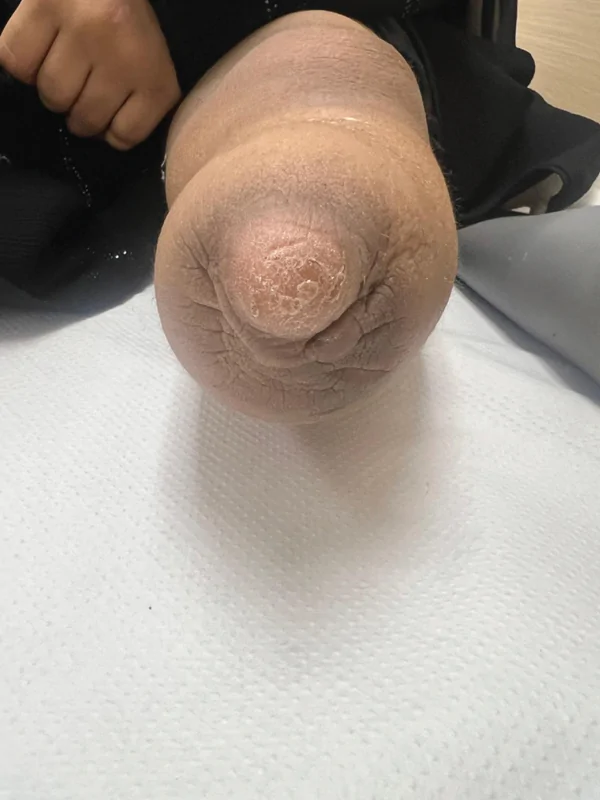
Clinical photograph of the left leg showing a distal osseous overgrowth causing skin pressure over the stump.

**Figure 2:**
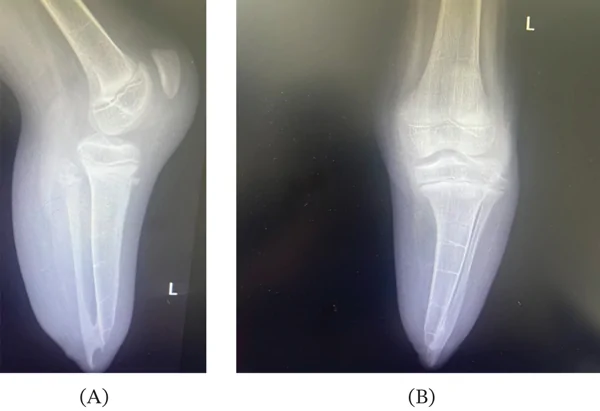
(A) Anteroposterior and (B) lateral radiographs of the left residual limb demonstrating below‐knee amputation with distal tibial and fibular osseous overgrowth.

The only anticipated issue as the patient continues to grow is recurrent osseous overgrowth, which may result in skin pressure, as shown in Figure 1. Radiographs demonstrated distal osseous overgrowth of the residual tibia and fibula (Figure 2). The patient and her family were counseled regarding the possibility of future stump revision procedures, and they demonstrated a good understanding of the expected course. Accordingly, she was scheduled for revision surgery.

**Figure 3:**
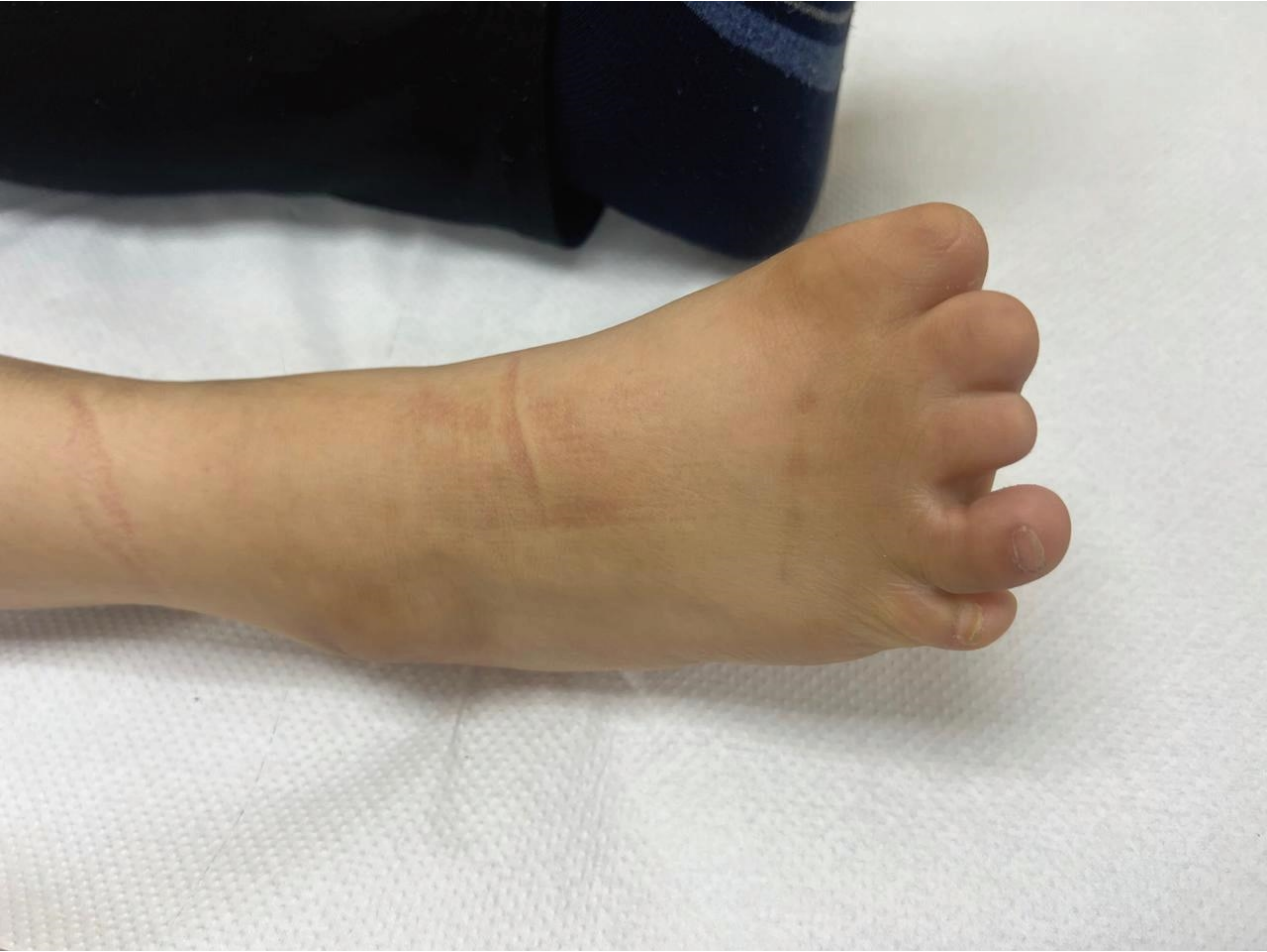
Clinical photograph of the right foot demonstrating maintained correction of congenital talipes equinovarus without recurrence. The associated digital anomalies are also visible.

**Figure 4:**
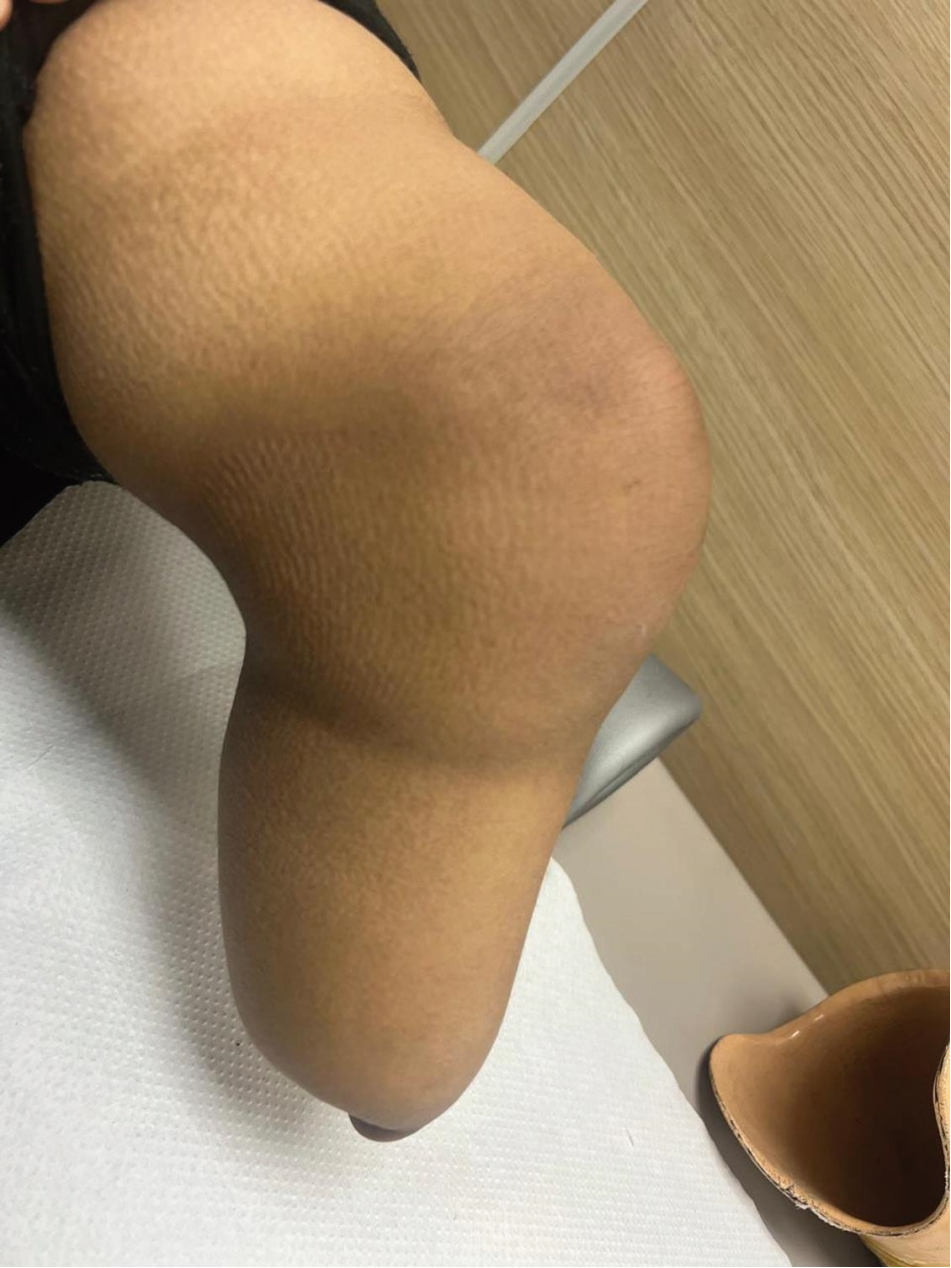
Clinical photograph of the left knee in flexion position and there is a mild band over the midthigh and a pressured stump caused by a growing bone.

**Figure 5:**
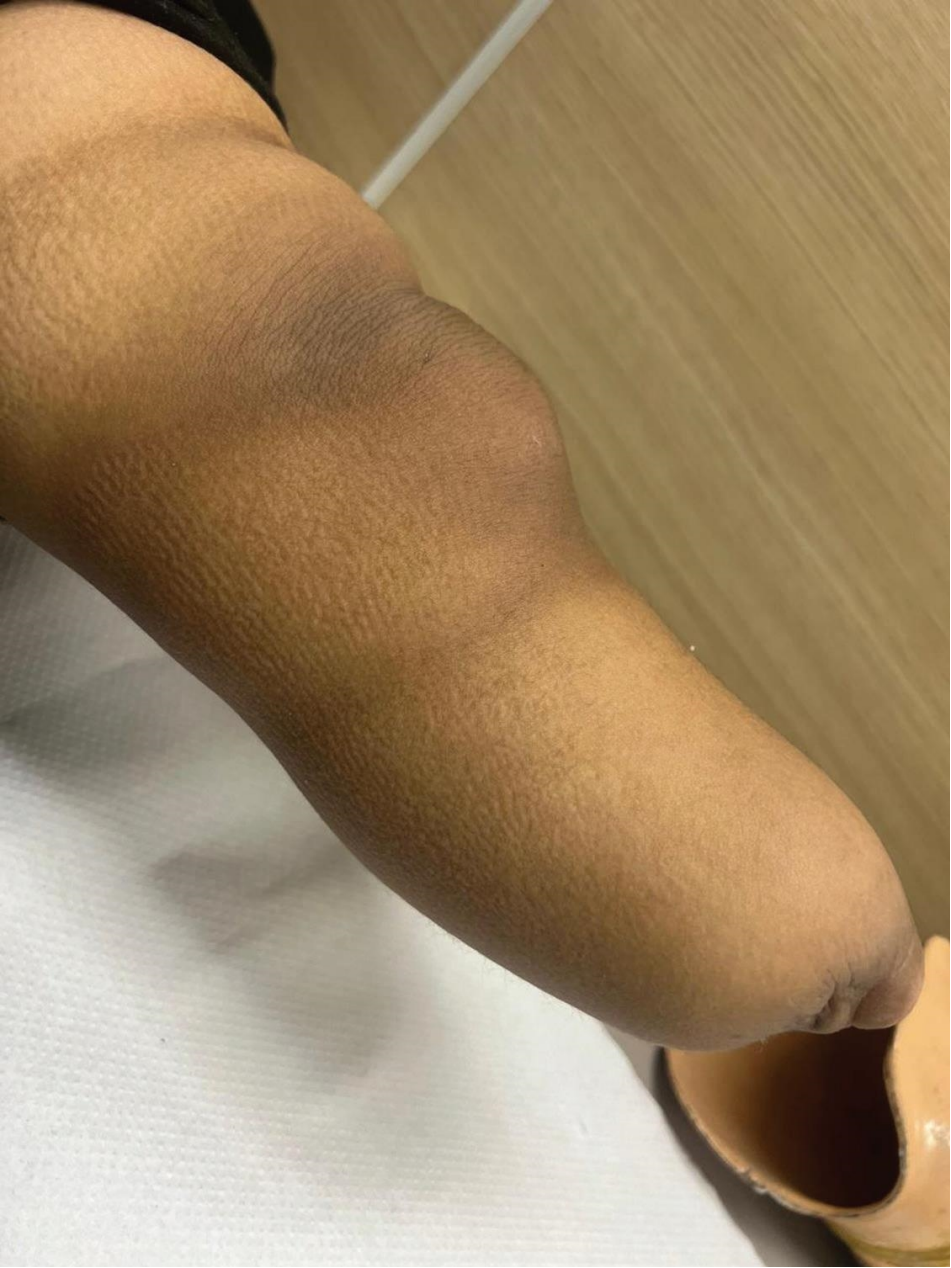
Clinical photograph of the left knee in extension position, and there is a mild band over the midthigh and a pressured stump caused by a growing bone.

**Figure 6:**
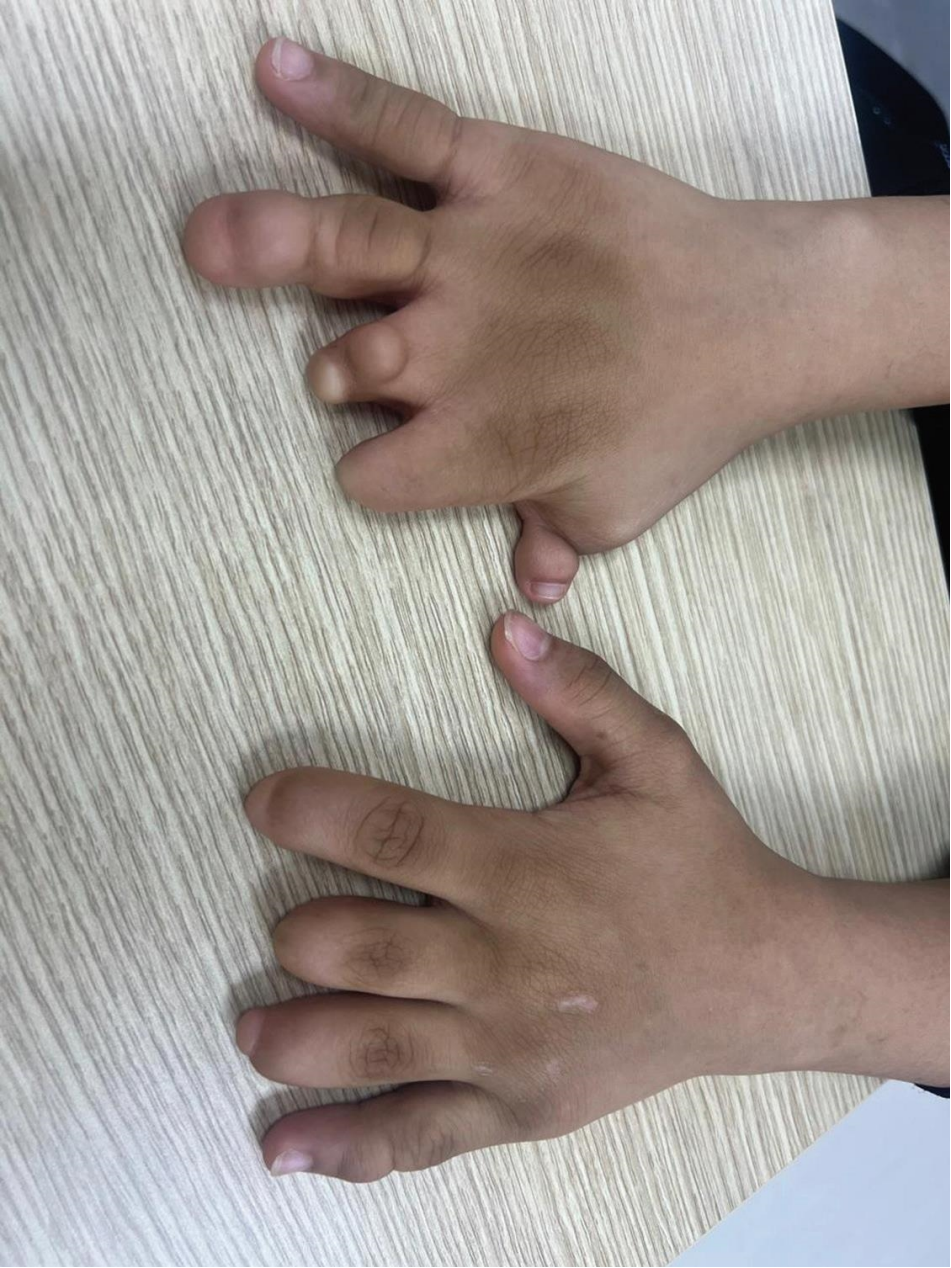
Clinical photograph of the bilateral hand showing congenital digital malformations and amputations (dorsal aspect).

**Figure 7:**
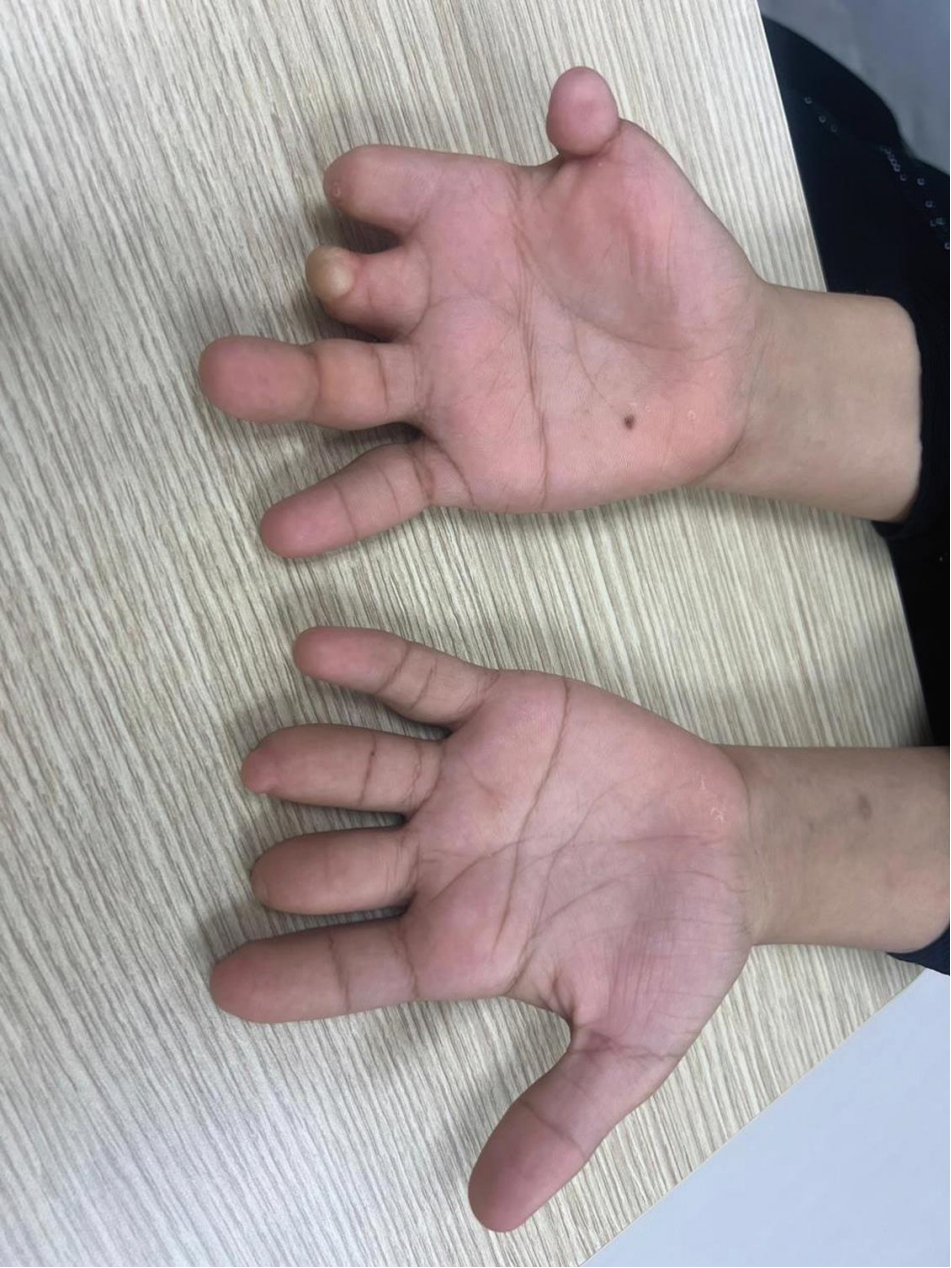
Clinical photograph of the bilateral hand showing congenital digital malformations and amputations (palmar aspect).

At her most recent outpatient clinic visit, the patient was walking independently with her prosthesis and demonstrated full weight bearing on the right lower limb. There were no signs of clubfoot recurrence, and the foot remained supple (Figure 3). The constriction ring on the left thigh had resolved spontaneously. The left knee range of motion was 0°–110° (Figures 4 and 5). Examination of both hands showed spontaneous resolution of the constriction band involving the left little finger, whereas the congenital digital hypoplasia remained unchanged from birth (Figures 6 and 7). She was able to write with a pen and perform fine motor tasks following rehabilitation training.

Overall, the patient is remarkably independent, thriving, well‐integrated socially, and actively engaged in her daily activities. She is performing excellently at school, has a good understanding of her condition, is psychologically well‐adjusted and resilient, and remains adherent to the multidisciplinary management plan.

## 3. Discussion

Streeter′s dysplasia, also known as ABS, is a collection of congenital malformations characterized by fibrous constriction bands that mainly affect the upper and lower limbs, craniofacial region, and thoracoabdominal area [1]. The reported incidence ranges from 1 in 1000 to 1 in 15,000 live births and approximately 178 per 10,000 spontaneous abortions, with an equal male‐to‐female ratio. Most cases are sporadic, with no associated genetic abnormalities or hereditary risk [3, 5].

Several theories have been proposed to explain the etiology of ABS, suggesting endogenous, exogenous, or genetic causes based on clinical observations and experimental studies [1]. In 1832, Montgomery proposed that intrauterine threads wrapped around the fetus, resulting in congenital deformities. Later, in 1930, Streeter suggested an endogenous theory, proposing that defective germ plasm differentiation leads to tissue necrosis and subsequent fibrous band formation [2]. However, the most widely accepted theory remains that described by Torpin in 1965, who proposed that rupture of the amniotic membrane allows fetal parts to pass into the chorionic cavity, where the developing fibrous bands progressively constrict the affected limbs or body parts [6].

Early prenatal diagnosis is achievable and facilitates close monitoring of the constricted fetal parts, allowing consideration of prenatal intervention in selected cases at specialized centers. Although US is routinely performed during pregnancy, some cases are not diagnosed until birth. US is typically performed between 10 and 12 weeks of gestation to evaluate craniofacial and thoracoabdominal anomalies, whereas limb abnormalities are more commonly detected between 20 and 22 weeks of gestation [1, 4, 5].

The clinical presentation of ABS ranges from mild constriction rings to severe deformities resulting in intrauterine amputation and may be associated with other orthopedic manifestations, including hypoplasia, syndactyly, CTEV, and leg length discrepancy. In our case, the patient was born with a left below‐knee amputation, right CTEV, and bilateral hand hypoplasia. Such complex presentations require coordinated multidisciplinary management involving surgical, rehabilitation, and medical teams [4].

Clubfoot is one of the most common orthopedic deformities associated with ABS and has been reported in approximately 49% of cases. Early management of CTEV is important to avoid delayed standing and walking. Most patients are successfully treated using the Ponseti serial casting protocol, including both correction and maintenance phases. However, the literature suggests that patients with ABS have a higher risk of recurrence and more resistant deformities [11–13]. Fortunately, our patient responded well to treatment, with no recurrence or need for further intervention during follow‐up.

Management of constriction bands depends on their severity. Mild constriction rings without neurovascular compromise may be managed conservatively, whereas more advanced cases may require one‐stage or two‐stage surgical release depending on the clinical presentation [5, 9].

Osseous overgrowth is an expected long‐term complication in pediatric amputees because bone growth exceeds the growth and stretching capacity of the surrounding soft tissues. If left untreated, it may lead to skin breakdown, ulceration, prosthetic intolerance, and infection. Management depends on the condition of the soft tissues and prosthetic tolerance and may involve prosthetic modification or surgical stump revision. However, recurrence is common, and repeated surgical excision may be required during growth [10, 14].

Rehabilitation and psychological support are essential components of long‐term management. Patient and family education, together with continuous multidisciplinary follow‐up, improves functional outcomes, enhances quality of life, and promotes adherence to the treatment plan [8].

The favorable functional outcome observed in our patient, together with her independence in daily activities, excellent school performance, and active social participation, highlights the importance of coordinated multidisciplinary care from the time of diagnosis through adolescence.

## 4. Conclusion

ABS is a congenital condition associated with a wide spectrum of malformations that may affect functionally important parts of the body. Understanding the clinical presentation of this syndrome and providing early intervention through a well‐structured multidisciplinary management plan are essential to optimize functional outcomes, improve quality of life, and maximize long‐term patient outcomes.

## Funding

No funding was received for this manuscript.

## Consent

Written informed consent was obtained from the patient′s legal guardian for publication of this case report and the accompanying clinical images.

## Conflicts of Interest

The authors declare no conflicts of interest.

## Data Availability

Data sharing is not applicable to this article as no datasets were generated or analyzed during the current study.
